# Applying nanopore sequencing technology in Paracoccidioides sp.: a high-quality DNA isolation method for next-generation genomic studies

**DOI:** 10.1099/mgen.0.001302

**Published:** 2024-10-21

**Authors:** Melina Noelia Lorenzini Campos, Ariel Fernando Amadio, José Matías Irazoqui, Raúl Maximiliano Acevedo, Florencia Dinorah Rojas, Luis Hernando Corredor Sanguña, Laura Belén Formichelli, Raúl Horacio Lucero, Gustavo Emilio Giusiano

**Affiliations:** 1 Instituto de Medicina Regional (IMR), Universidad Nacional del Nordeste (UNNE), Av. Las Heras 727, (3500) Resistencia, Chaco, Argentina; 2 Consejo Nacional de Investigaciones Científicas y Tecnológicas (CONICET), Godoy Cruz 2290, (C1425FQB) Ciudad Autónoma de Buenos Aires, Argentina; 3 Instituto de Investigación de la Cadena Láctea (IDICAL), Instituto Nacional de Tecnología Agropecuaria (INTA), Ruta 34 km 227, (2300) Rafaela, Santa Fe, Argentina; 4 Instituto de Botánica del Nordeste (IBONE, CONICET-UNNE), Universidad Nacional del Nordeste (UNNE), Sargento Juan Bautista Cabral 2131, (3402BKG) Corrientes capital, Argentina

**Keywords:** assembly, nanopore, *Paracoccidioides*, sequencing

## Abstract

Paracoccidioidomycosis is a severe systemic endemic mycosis caused by *Paracoccidioides* spp. which mainly affects individuals in Latin America. Progress in *Paracoccidioides* genomics has been slow, as evidenced by the incomplete reference databases available. Next-generation sequencing is a valuable tool for epidemiological surveillance and genomic characterization. With the ability to sequence long reads without the need for prior amplification, Oxford Nanopore Technology (ONT) offers several advantages, but high-quality and high-quantity DNA samples are required to achieve satisfactory results. Due to the low concentration of *Paracoccidioides* DNA in clinical samples and inefficient culture isolation methods, DNA extraction can be a significant barrier to genomic studies of this genus. This study proposes a method to obtain a high-coverage *de novo* genome assembly for *Paracoccidioides* using an improved DNA extraction method suitable for sequencing with ONT. The assembly obtained was comparable in size to those constructed from available data from Illumina technology. To our knowledge, this is the first genome assembly of *Paracoccidioides* sp. of such a large size constructed using ONT.

**Impact Statement**

This work represents an innovative approach as it allows the construction of a *de novo* genome assembly from third-generation Nanopore sequencing with a similar size to those obtained with Illumina technology. This is the first genomic assembly of *Paracoccidioides* sp. at such a large size made from this technology. Furthermore, it has the potential to significantly contribute to future sequencing efforts and the overall study of paracoccidioidomycosis by overcoming the challenges of limited DNA extraction from *Paracoccidioides* sp.

## Data Summary

Sample fastq files obtained by the authors of this work have been deposited at NCBI under the following accession numbers: SRA SRR30468353, Biosample SAMN39148619, Strain IMR-M-Pb 369, Taxonomy ID 3 110 790, BioProject PRJNA1057891. The IMR-M-Pb 369 genome project has been deposited at DDBJ/ENA/GenBank under the accession JBICBQ000000000. The version described in this paper is version JBICBQ010000000.

Raw data used for comparison were submitted by Christina Cuomo from the Broad Institute and are available at NCBI under the following accession numbers: SRA SRP077566, BioProjectPRJNA322632, Run SRR4024729, Biosample SAMN05171530 and Run SRR4024733, Biosample SAMN05171521.

All software used in this work is freely available online, mentioned in the Methods section and linked in the References. Additional information regarding the scripts, the method used to extract the DNA and the tree sequences can be found in the Supplementary Material, (available in the online version of this article).

## Introduction

Paracoccidioidomycosis is a severe systemic endemic mycosis caused by the fungal pathogen *Paracoccidioides* spp. which affects individuals predominantly in Latin America [1]. Unlike studies in *Saccharomyces*, *Candida*, *Aspergillus* and other fungi, progress in *Paracoccidioides* genomics has been slow and many details remain unknown. Its character as a temperature-dependent dimorphic fungus, presenting a filamentous fungal phase and a yeast phase, and the low viability of *Paracoccidioides* in culture are contributors to the difficulties encountered when working with this fungus. Furthermore, the lack of standardized tools and the incomplete reference databases available are additional factors that hinder progress [2].

The *Paracoccidioides* electrophoretic karyotype shows five chromosomal bands in the range 2.5–10 Mb, allowing an estimate of a total genome size of around 24–32 Mb [3]. Nevertheless, its low concentration in clinical samples and the inefficiency of current culture isolation methods [2] can present a significant challenge for genomic studies, as the requirements for DNA yield are greater than those for a PCR technique. Furthermore, studies in *Paracoccidioides* spp. have revealed that the genus possesses a thick cell wall [4,5], which represents a barrier to overcome to reach its DNA. Notwithstanding this, notable advancements have been made in the genome assemblies of reference strains through the utilization of Illumina’s short-read sequencing technology. This has ultimately resulted in the development of the current classification system for *Paracoccidioides* spp. and a more profound understanding of the genetic content and structural rearrangements as well as gene exchange, ploidy and speciation events [6,9].

As has been demonstrated, sequencing is a valuable tool for epidemiological monitoring and genomic characterization. Among the available technologies, Oxford Nanopore Technology (ONT) is considered a third-generation technology and offers several advantages by improving the rapid detection of pathogens, particularly those that cannot be identified by conventional methods or for which definitive typing has not been achieved. This technology has the ability to sequence long reads without the need for prior amplification. It is portable and provides real-time access to data [10]. However, to achieve quality results, it requires high-quality and high-quantity DNA samples. To our knowledge, there have been only two registered instances of genome sequencing using Nanopore technology for *Paracoccidioides* (SRA: SRP077566, BioProject: PRJNA322632), both with limited results.

Previous work using ONT in *Paracoccidioides* by Misas *et al*. [11] successfully sequenced and assembled the mitochondrial genome. The present study aims to contribute to future genomic studies of *Paracoccidioides* by proposing a method for obtaining a *de novo* genome assembly with high coverage using an improved DNA extraction method suitable for sequencing with Nanopore technology.

## Methods

The *Paracoccidioides* clinical isolate IMR-M-Pb 369 used in this study was obtained from a juvenile patient from the Northwest Argentine endemic region and subsequently deposited in the *Paracoccidioides* culture collection (IMR-M-Pb) at the Mycology Department, Instituto de Medicina Regional, Universidad Nacional del Nordeste, in Argentina.

The DNA extraction method suggested by Diez *et al*. [12] was improved with substantial modifications as briefly follows. In a class II biosafety cabinet, 0.3 g of yeast biomass was suspended in 500 µl TES solution (0.1 M Tris/HCl pH 8, 10 mM EDTA, 2% SDS) and vortexed with 0.3 g of glass beads (850–425 µm) for 15 min. Treatment with 10 µl Proteinase K (10 mg ml^−1^) was carried out for 1 h at 55 °C. After centrifugation at 12 000 r.p.m. for 1 min, supernatant was transferred into a new tube with 500 µl CTAB solution (1.4 M NaCl, 20 mM EDTA, 100 mM Tris/HCl pH 8.0, 0.2% 2-mercaptoethanol, 5% CTAB) and incubated at 65 °C for 1 h. A first extraction was made with 500 µl chloroform/isoamyl alcohol (24 : 1). After recovery of the supernatant, treatment with 5 µl RNAse A (10 mg ml^−1^) was applied at 37 °C for 30 min, followed by a second extraction with 500 µl chloroform/isoamyl alcohol (24 : 1). DNA precipitation was achieved by overnight incubation at −20 °C, after adding 2.5 volumes of pure ethanol. After centrifugation at 12 000 r.p.m. for 15 min, the pellet was recovered and washed twice with 500 µl ethanol (70%). Finally, DNA was resuspended in 60 µl of molecular biology-grade water and analysed with a Nanodrop device (Thermo Fisher Scientific). DNA integrity was visualized in an agarose (1%) gel. To corroborate reproducibility, the extraction process was repeated three times. The detailed method is available in the Supplementary Material.

Prior to sequencing, DNA molecules were concentrated and further purified with magnetic beads (1 : 2). Then the sample was measured with a dsDNA kit on a Qubit (Thermo Fisher Scientific). Library preparation started with 1 µg DNA, following the Nanopore ‘Genomic DNA by Ligation (SQK-LSK109)’ protocol, without a fragmentation step. Sequencing was performed on a MinION Mk1C MIN 101 C, with a FLO-MIN106D R9 flow cell (ONT), according to the manufacturer’s instructions. Reads were basecalled using dorado (v7.1) [13], with the super high-accuracy model. All low-quality (Q<10) and short reads (<500 bp) assessed by PycoQC (v2.5.2) [14] were filtered out using FiltLong [15]. An assembly was obtained using Flye (2.9.4) [16] and polished using Medaka (v1.12.1) [17]. All reads were mapped against the resulting contigs using Minimap2 (2.28) [18] and samtools (1.17) [19]. To assess the quality and completeness of the assembly, the contigs were compared against a genomic reference assembly (GCF_000150735.1, https://www.ncbi.nlm.nih.gov/datasets/genome/GCF_000150735.1/) [20] and a mitochondrion reference assembly (MT815704.1, https://www.ncbi.nlm.nih.gov/nuccore/MT815704.1/) [11] using blast+ [21]. Genomic hits were used to verify the completeness of the five chromosomes, using genoplotR [22] and Rstudio [23]. For the mitochondrion, all reads mapping to contigs matching the reference were used to reassemble its genome. Since the estimated coverage of the mitochondrion was excessively high, reads were filtered to ensure an estimated 150× coverage using FiltLong and assembled using Flye.

A phylogenetic tree of the GP43 gene was used, as it has been previously used to classify subspecies in the genus *Paracoccidioides* [24,25]. Reference sequences were obtained from different previous reports (Supplementary Material) and compared to assembled contigs using blast+ to identify the region of interest. All sequences were assembled using MAFFT (v7.409) [26] and a phylogenetic tree was reconstructed using RAxML [27], with the GTR-GAMMA-i model and 1000 bootstraps. The figure was obtained using iTOL [28].

In parallel, statistics obtained with Assemblathon [29] were compared with those performed on assemblies made with SPAdes-3.15.5 [30] from raw data of two different biosamples available in the NCBI database: S1a_PbER and S1b_PbCAZ (SRA: SRP077566, BioProject:PRJNA322632, Run: SRR4024729, Biosample: SAMN05171530 and Run: SRR4024733, Biosample: SAMN05171521, both from Illumina Technology, submitted by Christina Cuomo from the Broad Institute at https://www.ncbi.nlm.nih.gov/Traces/study/?acc=SRP077566&o=instrument_s%3Ad) [31]. Further evaluation of the assemblies was performed with BUSCO completeness (v5.7.1) [32] and AntiSMASH 7.0 fungal version [33].

Two previous attempts at sequencing *Paracoccidioides* using Nanopore technology are available in the NCBI database (Run SRR12032017, Biosample SAMN05171520 and Run SRR12032016, Biosample SAMN05171542). However, they were unable to yield sufficient data to represent the complete genome, and thus were discarded.

## Results

The improved isolation method produced high-quality and high-quantity DNA as required for the Nanopore sequencing technology. DNA concentrations exceeding 1000 ng µl^–1^ and *A*_260/280_ ratio between 1.8 and 2 were observed with Nanodrop.

A total of 729 298 reads were obtained, representing 1.453 Gb, of which approximately 60% remained after quality control (290 053 reads, representing 0.88 Gb). Fastq files have been deposited at NCBI under the following accession numbers: SRA SRR30468353, Biosample SAMN39148619, Strain IMR-M-Pb 369, Taxonomy ID 3 110 790, BioProject PRJNA1057891.

The IMR-M-Pb 369 genome assembly reached a size of 28865944 bp, consisting of 59 contigs with N50=2133236, L50=4 and a coverage of 25×. Contigs larger than 1 Mb were also obtained. In addition, the mitochondrial genome was recovered in one contig of 117 633 bp. This genome project has been deposited at DDBJ/ENA/GenBank under the accession JBICBQ000000000. The version described in this paper is version JBICBQ010000000.

**Fig. 1.**
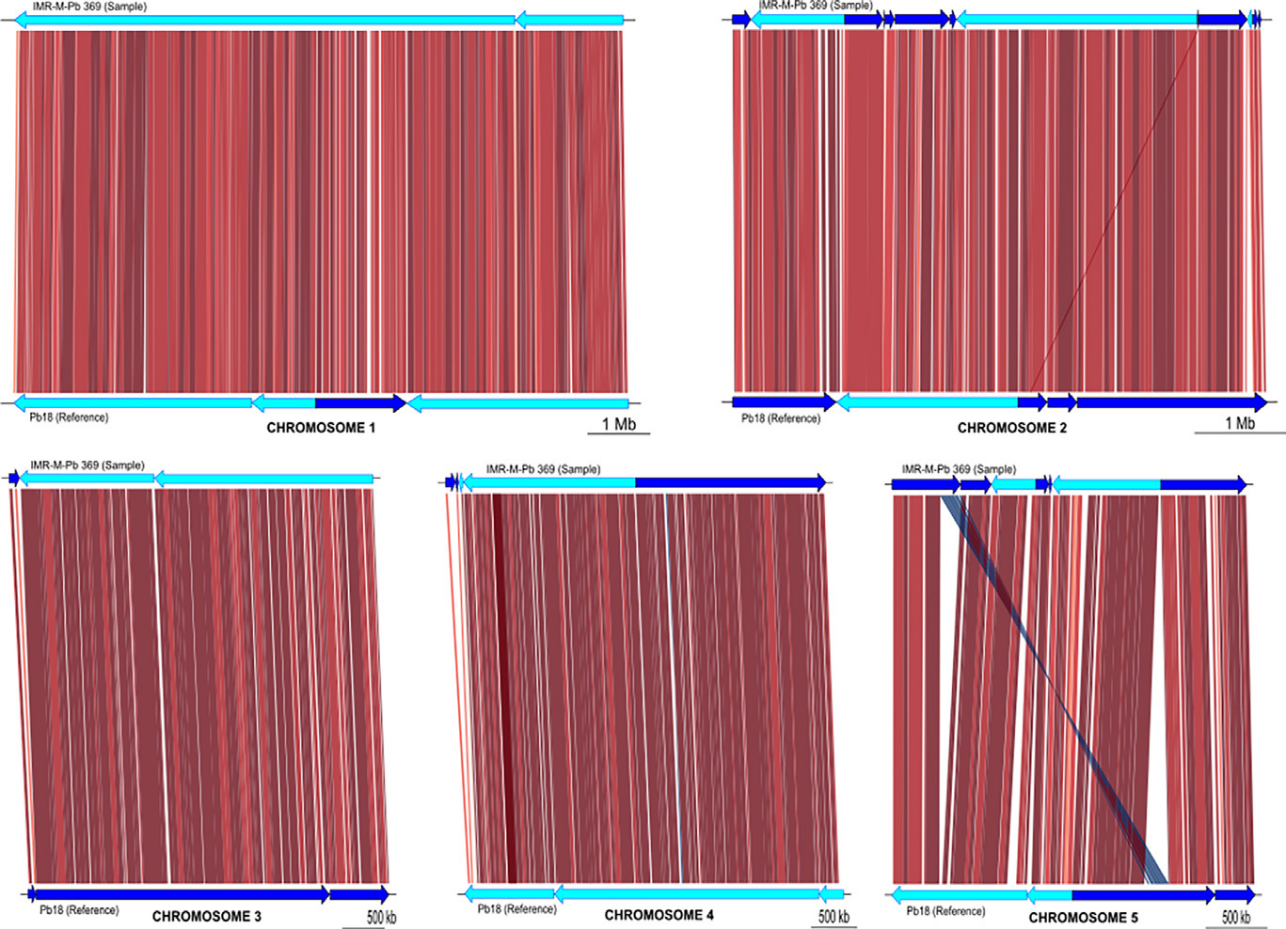
Schematic representation of the alignment of *Paracoccidioides* isolate IMR-M-Pb 369 sequences to the five chromosomes of reference Pb18 [20]. The isolate sequences (sample) are shown at the top of each graph, with the references at the bottom. The orientation of the fragments is indicated by dark and light blue arrows. The inversion identified in chromosome 5 of isolate IMR-M-Pb 369 is highlighted.

All chromosomes in IMR-R-Pb 369 showed a high degree of structural conservation when compared to Pb18, except for chromosome 5, where one of the contigs assembled (contig 23) matched with two different contigs from the reference, contigs 10 and 8. These two contigs were placed far apart in the optical map presented by Desjardins *et al*. [20]. Also, contig 23 in our assembly showed a 173 650 bp inversion related to contig 8 from Pb18. A schematic representation of the five chromosome alignments is displayed in Fig. 1.

Comparison between the IMR-M-Pb 369, S1a_PbBER and S1b_PbCAZ assemblies resulted in similar sizes but with a significantly reduced number of contigs in our isolate (Table 1).

**Table 1.** Statistical comparisons of the assemblies obtained from isolate IMR-M-Pb 369 and from the raw data available in the NCBI database SRA: SRP077566, BioProject: PRJNA322632

Assemblathon_stats	Isolate IMR-M-Pb 369 (Nanopore)	S1a_PbBER (Illumina)	S1b_PbCAZ (Illumina)
Number of contigs	59	3 596	3 234
Total size of contigs (bp)	28865944	29 847 218	29 795 588
Longest contig (bp)	8300986	423 526	314 969
Shortest contig (bp)	1719	78	78
Number of contigs > 1Kbp	59 (100.0%)	601 (16.7%)	706 (21.8%)
Number of contigs > 10Kbp	46 (78.0%)	459 (12.8%)	518 (16%)
Number of contigs > 100Kbp	24 (40.7%)	78 (2.2%)	70 (2.2%)
Number of contigs > 1Mbp	8 (13.6%)	0	0
Mean contig size (bp)	489253	8300	9213
Median contig size (bp)	51 706	146	170
N50 contig length (bp)	2133236	85 985	72 628
L50 contig count	4	106	129

In terms of expected gene content of the assemblies related to 4862 markers of the Onygenales dataset, BUSCO yielded the following results: for isolate IMR-M-Pb from Nanopore, 89.4% complete and single-copy (CS), 0.1% complete and duplicated (CD), 5.8% fragmented (F) and 4.7% missing (M) BUSCOs. In the case of the assemblies made from Illumina, both exhibited 96.6% CS, 0% D, 0.9% F and 2.5% M with the following differences: 42 F and 123 M for S1a_PbBER while 44 F and 121 M for S1b_PbCAZ. Moreover, the AntiSMASH fungal version facilitated the identification of regions associated with type I polyketide synthases (T1PKS), beta-lactone-containing protease inhibitors, non-ribosomal peptide synthetases (NRPS) and terpenes in the three assemblies through a strict detection approach. Furthermore, in a relaxed detection mode, fungal RiPP-like matches related to transport genes and NRPS-like fragments were also identified.

**Fig. 2.**
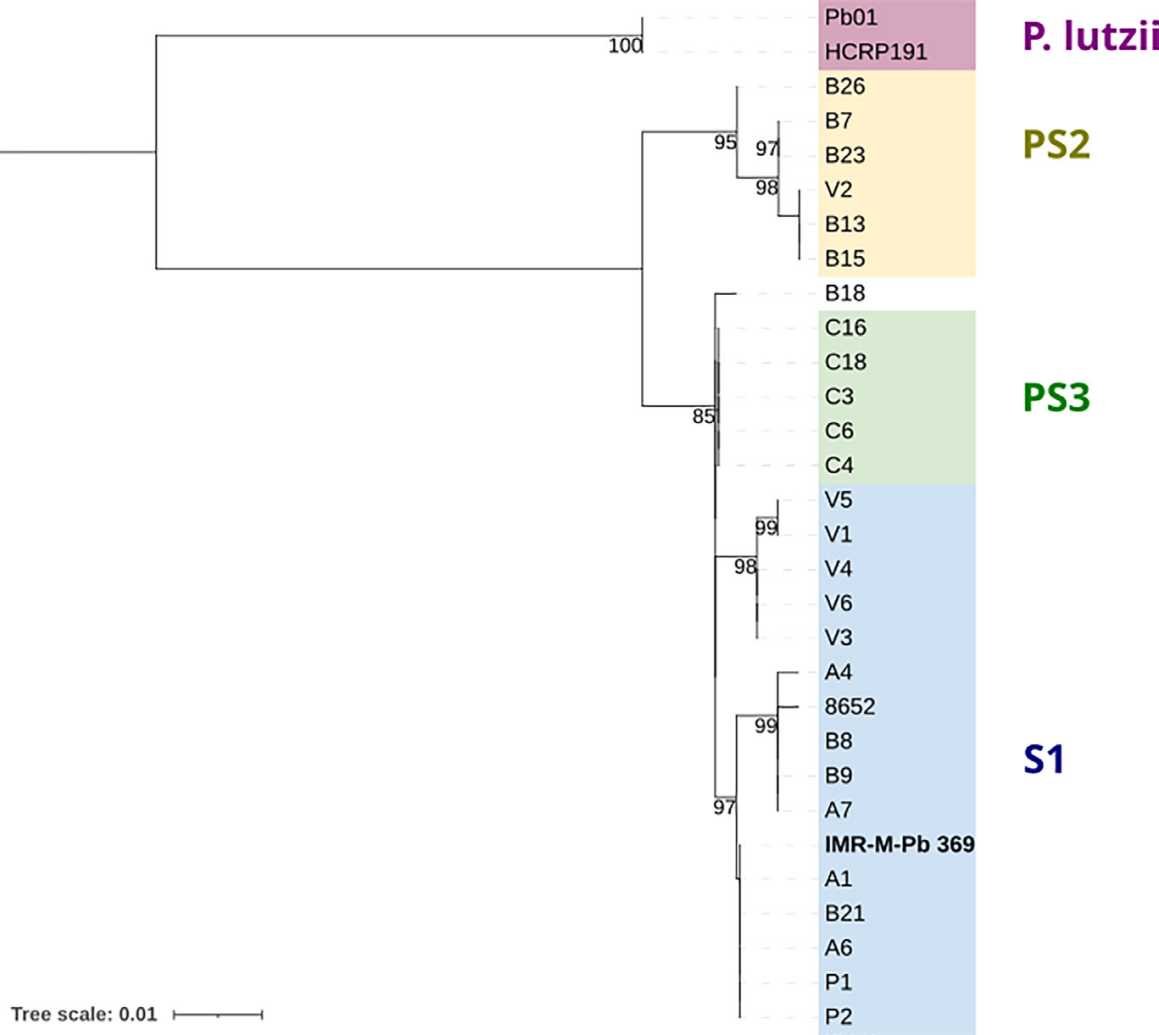
Phylogenetic tree based on exon 2 of the GP43 gene, according to previous studies [24,25]. Isolate IMR-M-Pb 369 sequenced with Nanopore technology in the present study is highlighted. Numbers on the branches indicate bootstrap values. Scale bar refers to a phylogenetic distance of 0.01 nucleotide substitutions per site.

To attempt an orientative classification with the sequencing information available from our isolate and in light of the limitations of original Nanopore chemistry in terms of accuracy in the detection of SNPs, the sequence of exon 2 of the GP43 gene was used to reconstruct a phylogenetic tree in accordance with previous studies [24,25]. The resulting tree is presented in Fig. 2, which illustrates that our isolate appears to be more closely related to the *Paracoccidioides brasiliensis* species classified as S1.

## Discussion

Although the detailed DNA extraction protocol published by Diez *et al*. [12] is appropriate for conducting PCR techniques, it is essential for ONT sequencing to achieve the optimal DNA quality yield in order to fully capitalize on the potential of this innovative technology. In this regard, our updated isolation method ensured the provision of an appropriate input, thus meeting the necessary requirements for successful sequencing. The adjusted mechanical disruption step played a key role in increasing the yield of extracted DNA, which may have contributed to the breakdown of the thick cell wall of *Paracoccidioides* [4]. The tailored subsequent purification step was important to reach the quality parameters while the use of magnetic beads allowed further concentration of the DNA molecules. Although this study focused on the use of Nanopore technology, which has strict requirements for the sample to be sequenced, we believe that the proposed DNA extraction method could also be useful for sequencing with other technologies as well.

The sequencing process of *Paracoccidioides* using ONT was successful, resulting in a *de novo* assembly of a similar size to those constructed from Illumina technology, both by us in this work with data available in NCBI (SRA: SRP077566, BioProject: PRJNA322632) and by other authors [7]. Statistical comparisons of the assemblies showed that ONT achieved the goal with a small number of contigs, some of which reached 1 Mb. It also allowed the recovery of the mitochondrial genome, with very high coverage, in one contig, similar in size to previous attempts [11]. This is attributed to the ability of the Nanopore technology to sequence long reads, which is also evident in the N50 and L50 values. This advantage can also be useful in reconstructing chromosomes, such as the one in this study, as shown in the schematic representation of the mapping against the S1b Pb18 reference sequences [20]. Interestingly, our assembly showed a portion of the genome displaced and inverted when compared to the reference genome Pb18. Although a deeper analysis of the genes encoded in this region is needed, the identification of these rearrangements was only possible because a *de novo* assembly strategy was used.

With regard to the classification of *Paracoccidioides* species, a variety of approaches have been employed historically, with different results dependent on the technology used [24,34]. To illustrate this, strain Pb18, for which the optical map was used in this study for alignment (Fig. 1), is currently typified as S1b, whereas it was classified as S1 when the optical map was first released [20]. In the absence of data derived from Illumina sequencing of our isolate IMR-M-Pb 369 and given the limitations of Nanopore’s original chemistry in single nucleotide polymorphism (SNP) detection accuracy, we opted to extract the sequence of exon 2 of the GP43 gene from the genomic assembly and use it to reconstruct a phylogenetic tree in accordance with previous studies [24,25, 35]. The GP43 gene encodes the 43 kDa glycoprotein, which is representative of the genus. Studies such as the one conducted by Cocio *et al*. [36] have aimed to establish a correlation between the results obtained from this gene approach and the most recent classification of *Paracoccidioides* species based on Illumina sequencing. As can be observed in our phylogenetic tree, our isolate appears to be more probably closely related to the species classified as *P. brasiliensis* S1. However, as the results of any classification attempt depend on the specific fragment examined, and considering that the current classification of the genus is based on the analysis of SNPs from whole-genome sequencing (WGS) using Illumina technology, we advocate supplementing the present study with a more comprehensive coverage of the recently available ONT sequencing chemistry to improve the accuracy of SNP detection [37,38], which is of concern for WGS phylogenetic analysis.

It is widely acknowledged that fungal genomes are significantly larger and more complex than those of viruses and bacteria. This implies the need to address a greater number of chromosomes and introns as well as to consider mitochondrial sets. Sequencing the fungal genome using Nanopore technology can be a resource-intensive process, as well as involve considerable time and server capacity for the necessary bioinformatic processing and analysis of the resulting data. This certainly poses a challenge for the vast majority of clinical laboratories in Latin America that receive patients with paracoccidioidomycosis. However, it is important to acknowledge the potential benefits of this approach. It may be worth considering the establishment of stronger networks as a means to serve this purpose.

The sequencing and analysis of the genome of the genus *Paracoccidioides* plays a critical role in advancing our understanding of its biology, pathogenicity and epidemiology. This information is crucial for improving the diagnosis and treatment of this endemic mycosis caused by a fungus included in the list of priority pathogens issued by the World Health Organization (WHO) in 2022 [39]. As the genus *Paracoccidioides* comprises multiple species with notable genomic variations, their sequencing will enable the investigation of these discrepancies, thereby providing a deeper understanding of speciation, geographical distribution and the pathogen’s evolutionary trajectory. This is of paramount importance for the implementation of more efficacious public health strategies, a common deficiency in Latin America [40]. Furthermore, genomic analysis is vital for the identification of genes associated with pathogenicity. This is fundamental to understand how this fungus establishes infection, invades tissues and causes damage, which may lead to the development of new therapeutic strategies and the identification of novel targets for effective antifungal drugs. As one of the major challenges with this disease is the lack of rapid detection tools [2], genomic sequencing can assist in the identification of specific molecular markers that could be employed in the development of new, sensitive diagnostic methods and the implementation of easily accessible reading systems. Ultimately, genomic studies contribute to the identification of immunogenic proteins or peptides that could serve as potential candidates for the development of vaccines or more specific serological tests for this mycosis, for which currently available preventive measures are lacking.

It is our belief that our proposal has the potential to make a significant contribution to future sequencing efforts of *Paracoccidioides* spp. The ability of Nanopore technology to work with long reads is especially beneficial for the discovery of structural rearrangements, which have a profound impact on previously described targets in the broader context of paracoccidioidomycosis research.

## Conclusions

The proposed method of DNA isolation in *Paracoccidioides* sp. was suitable for genomic sequencing studies using Nanopore technology, which requires very high-quality and high-quantity DNA samples. ONT allowed a *de novo* assembly with a similar size to those developed from available data obtained using Illumina technology. To our knowledge, this is the first genomic assembly of *Paracoccidioides* sp. of such large size made from ONT.

## supplementary material

10.1099/mgen.0.001302 Uncited Supplementary Material 1.

## Abbreviations

CD: complete and duplicated
CS: complete and single-copy
F: fragmented
M: missing
NRPS: non-ribosomal peptide synthetase
ONT: Oxford Nanopore Technology
RiPPs: ribosomally synthesized and post-translationally modified peptides
SNP: single nucleotide polymorphism
T1PKS: type I polyketide synthase
WGS: whole genome sequencing
WHO: World Health Organization
