# Supplementary material for: Applying nanopore sequencing technology in Paracoccidioides sp.: a high-quality DNA isolation method for next-generation genomic studies
Source: Microb Genom. 2024 Oct 21;10(10):001302. doi: 10.1099/mgen.0.001302 (PMC11493184; doi:10.1099/mgen.0.001302)
Supplement: Uncited Supplementary Material 1. [file mgen-10-01302-s001.pdf]

Supplementary Material

- DNA extraction from yeast culture of *Paracoccidioides spp*

WARNING: Always work in a class II biosafety cabinet.
NOTE: All centrifugation steps are carried out at room temperature.

1. In a class II biosafety cabinet, resuspend 0.3 g of a pure yeast culture in a 2 ml screw cap tube with 500 µl of TES solution (0.1 M Tris-HCl pH 8; 10 mM EDTA; 2% SDS) and 0.3 g of glass beads (850-425 µm).
2. Vortex for 15 minutes.
3. Add 5µl of Proteinase K (10 mg/ml). Incubate at 55 °C for 1 hour.
4. Centrifuge at 12000 rpm for 1 minute and transfer the supernatant into a new tube cautiously to avoid carrying away cellular debris from the pellet.
5. Add 500 µl of 2% CTAB solution (1.4 M NaCl; 20 mM EDTA; 100 mM Tris-HCl pH 8; 0.2 % 2-Mercaptoethanol; 5 % CTAB). Incubate at 65 °C for 1 hour.
6. Add 500 µl of chloroform:isoamyl alcohol (24:1) and mix by inverting the tube until the phases are completely mixed.
7. Centrifuge at 12000 rpm for 5 minutes. Recover the supernatant (aqueous phase) into a new tube. Avoid pipetting and transferring the interface and the lower organic phase.
8. Add 5µl of Rnase A (10mg/ml). Incubate at 37 °C for 30 minutes.
9. Repeat steps 6 and 7 once.
10. To precipitate the DNA, add 2.5 volumes of -20ºC stored pure ethanol and mix gently. Incubate at -20 °C overnight or at least for 30 minutes.
11. Centrifuge at 12000 rpm for 15 minutes. Carefully discard the supernatant without disturbing the pellet.
12. Add 500µl of cold 70% ethanol.
13. Centrifuge at 12000 rpm for 5 minutes. Discard the supernatant. Carefully discard the supernatant without disturbing the pellet.
14. Repeat steps 12 and 13 once.
15. Dry 30 minutes at 50ºC.
16. Resuspend the pellet in 60µl of molecular biology grade water or in TE buffer.

- Scripts

Basecalling, reads filtering and assembly

```
$ dorado basecaller --emit-fastq dna_r9.4.1_e8_sup@v3.3 paracocci.pod5 > paracocci.fastq
```

```
$ filtlong --min_mean_q 10 --min_length 1000 paracocci.fastq > $ paracocci.500_10q.fastq
```

```
$ flye --nano-hq paracocci.500_10q.fastq --threads 55 -o assembly
```

```
$ medaka_consensus -d assembly/assembly.fasta -i paracocci.500_q10.fastq.gz -t 40 -m r941_min_sup_g507
```

All contigs with length below 1000bp were removed

Chromosome and mitochondrion classification

```
$ blastn -query paracocci_IMR-M-Pb_369.fasta -subject Paracocci_br_Pb01_V2_genomic.fna -outfmt 6 -out chromosome.blastn
```

```
$ blastn -query paracocci_IMR-M-Pb_369.fasta -subject Paracoccidiodes_brasiliensis_mitochondrion.fna -outfmt 6 -out mitochondrion.blastn
```

All IDs of contigs matching with mitochondria are extracted from the blasts results and stored in the file "mitochondrian_contigs.list"

Mitochondrion re-assembly

```
$ minimap2 -x map-ont -t 55 -a -o paracocci_IMR-M-Pb_369.sam paracocci_IMR-M-Pb_369.fasta paracocci.fastq
```

```
$ grep -f mitochondrion_contigs.list paracocci_IMR-M-Pb_369.sam | cut -f 1 > mitochondrion_reads.list
```

```
$ grep -f mitochondrion_reads.list -A 3 --no-group-separator paracocci.500_10q.fastq > mitochondrion_reads.fastq
```

```
$ filtlong -t 17649600 mitochondrion_reads.fastq > mitochondrion_reads.150X.fastq
```

```
$ flye --nano-hq mitochondrion_reads.150X.fastq --threads 40 -o mitochondrion_assembly
```

```
$ medaka_consensus -d mitochondrion_assembly/assembly.fasta -i mitochondrion_reads.150X.fastq -t 40 -m r941_min_sup_g507
```

Phylogeny

```
$ blastn -query paracocci_IMR-M-Pb_369.fasta -subject GP43_Pb01.fasta -outfmt 6 -out GP43.blastn
```

The gene is extracted from the assembly using BLAST's match coordenates and one file with all the GP43 seqeunces available is constructed

```
$ cd-hit -c 1 -i GP43.fasta -o GP43.reduced
```

```
$ mafft --maxiterate 1000 --localpair --thread 55 GP43_reduced.fasta > GP43.aligned.fasta
```

```
$ raxmlHPC-PTHREADS-SSE3 -m GTRGAMMAI -n GP43 -p 123654 -s GP43.aligned.fasta -T 50 -# 20
```

```
$ raxmlHPC-PTHREADS-SSE3 -m GTRGAMMAI -n GP43_bootstraps -p 123654 -s GP43.aligned.fasta -T 50 -N 1000 -x 123654
```

```
$ raxmlHPC-PTHREADS-SSE3 -m GTRGAMMAI -p 123654 -f b -t RAxML_bestTree.GP43 -z RAxML_bootstrap.GP43_bootstraps -n GP43_final
```

- <u>Phylogenetic tree sequences (exon 2 of GP43)</u>

Strain	Species	Accession	Reference
B13	P. brasiliensis PS2	DQ003736.1	Matute et al., 2006
B15	P. brasiliensis PS2	DQ003738.1	Matute et al., 2006
B23	P. brasiliensis PS2	DQ003746.1	Matute et al., 2006
B26	P. brasiliensis PS2	DQ003749.1	Matute et al., 2006
B7	P. brasiliensis PS2	DQ003730.1	Matute et al., 2006
V2	P. brasiliensis PS2	DQ003772.1	Matute et al., 2006
C3	P. brasiliensis PS3	DQ003752.1	Matute et al., 2006
C4	P. brasiliensis PS3	DQ003753.1	Matute et al., 2006
C6	P. brasiliensis PS3	DQ003755.1	Matute et al., 2006
C16	P. brasiliensis PS3	DQ003765.1	Matute et al., 2006
C18	P. brasiliensis PS3	DQ003767.1	Matute et al., 2006
8652	P. brasiliensis S1	KY963815.1	Hrycyk et al. (2018)
A1	P. brasiliensis S1	DQ003780.1	Matute et al., 2006
A4	P. brasiliensis S1	DQ003781.1	Matute et al., 2006
A6	P. brasiliensis S1	DQ003783.1	Matute et al., 2006
A7	P. brasiliensis S1	DQ003784.1	Matute et al., 2006
B8	P. brasiliensis S1	DQ003731.1	Matute et al., 2006
B9	P. brasiliensis S1	DQ003732.1	Matute et al., 2006
B21	P. brasiliensis S1	DQ003744.1	Matute et al., 2006
P1	P. brasiliensis S1	DQ003786.1	Matute et al., 2006
P2	P. brasiliensis S1	DQ003787.1	Matute et al., 2006
V1	P. brasiliensis S1	DQ003771.1	Matute et al., 2006
V3	P. brasiliensis S1	DQ003773.1	Matute et al., 2006
V4	P. brasiliensis S1	DQ003774.1	Matute et al., 2006
V5	P. brasiliensis S1	DQ003775.1	Matute et al., 2006
V6	P. brasiliensis S1	DQ003776.1	Matute et al., 2006
HCRP191	P. lutzii	MK909806.1	Cocio et al., 2020
Pb01	P. lutzii	EU870196.1	Teixeira et al., 2009
